# Editorial: The role of inflammation and immune control in digestive disease and therapeutic approaches

**DOI:** 10.3389/fimmu.2025.1621130

**Published:** 2025-05-16

**Authors:** Zhaoyang Wang, Shuai Wang

**Affiliations:** ^1^ Huadu District People’s Hospital of Guangzhou, Huadu Institute of Medicine, Guangzhou, China; ^2^ State Key Laboratory of Traditional Chinese Medicine Syndrome, School of Pharmaceutical Sciences, Guangzhou University of Chinese Medicine, Guangzhou, China; ^3^ R&D Department, Chinese Medicine Guangdong Laboratory, Hengqin, China

**Keywords:** digestive tract diseases, inflammatory bowel disease, therapeutic targets, immunity, inflammation

In recent years, digestive tract diseases have posed an increasingly serious threat to global health. Conditions such as inflammatory bowel disease (IBD), gastroesophageal reflux disease (GERD), and irritable bowel syndrome (IBS) are becoming more common and are closely associated with persistent inflammation and disrupted immune regulation. Taking IBD as an example, it serves as a representative immune-mediated digestive disease. Commonly used treatments such as aminosalicylates, corticosteroids and immunomodulators can relieve symptoms to some extent, but their effectiveness is often limited. Long-term use is frequently accompanied by drug resistance and considerable side effects. These challenges highlight the urgent need to better understand the immunological and inflammatory mechanisms underlying these conditions. Advancing this understanding is essential for identifying new therapeutic targets and developing more precise interventions to manage digestive tract diseases and restore immune balance.

Advances in molecular biology and immunology have led to the identification of key targets involved in the inflammatory and immune dysregulation that characterize digestive tract diseases. In IBD, aberrant expression of genes such as CXCR4, THY1, CCL20, and CD2 has been implicated in disease progression. These molecular changes are accompanied by the infiltration and dysfunction of immune cell populations, including Th17 cells, regulatory T cells (Tregs), and neutrophil extracellular traps (NETs), which represent hallmark features of intestinal inflammation (Liu et al., Long et al.). Among critical pathways, the IL-6/JAK2/STAT3 axis plays a central role in orchestrating inflammatory responses and T cell differentiation. Dysregulated activation of this pathway promotes excessive production of pro-inflammatory cytokines such as IL-6 and IL-17, and disrupts the Th17/Treg balance, thereby amplifying mucosal inflammation (Tang et al.). This Research Topic also revealed that miR-146a serves as a negative regulator of inflammation by inhibiting the NF-κB signaling cascade and downstream pro-inflammatory gene expression. Reduced miR-146a expression is closely associated with heightened inflammatory responses (Zhu et al.). Moreover, circadian clock component BMAL1 has been shown to regulate autophagy and maintain epithelial barrier integrity by sustaining the expression of tight junction proteins, including Claudin-1 and ZO-1. Loss of BMAL1 function compromises these protective mechanisms, contributing to barrier dysfunction (Zhang et al.). Furthermore, overactivation of the PI3K/AKT signaling pathway induces apoptosis in intestinal epithelial cells (IECs) by upregulating pro-apoptotic proteins including Bax and Caspase-3, whereas targeted inhibition of this pathway contributes to the preservation of barrier function (Huang et al.). Beyond IBD, distinct molecular mechanisms have been identified in other digestive disorders. In GERD, epithelial injury is not solely driven by acid exposure but also involves activation of the NLRP1/Caspase-1/GSDMD pyroptotic pathway (Kim et al.). In IBS, abnormal activation of Toll-like receptor 4 (TLR4) and loss-of-function mutations in nucleotide-binding oligomerization domain 2 (NOD2) contribute to the disorder’s complex immune and microbial interactions (Wan et al., Masaki et al.). Collectively, these findings outline a multifaceted molecular framework underlying the immune and inflammatory dysregulation in digestive tract diseases, offering potential therapeutic targets for intervention.

**Figure 1:**
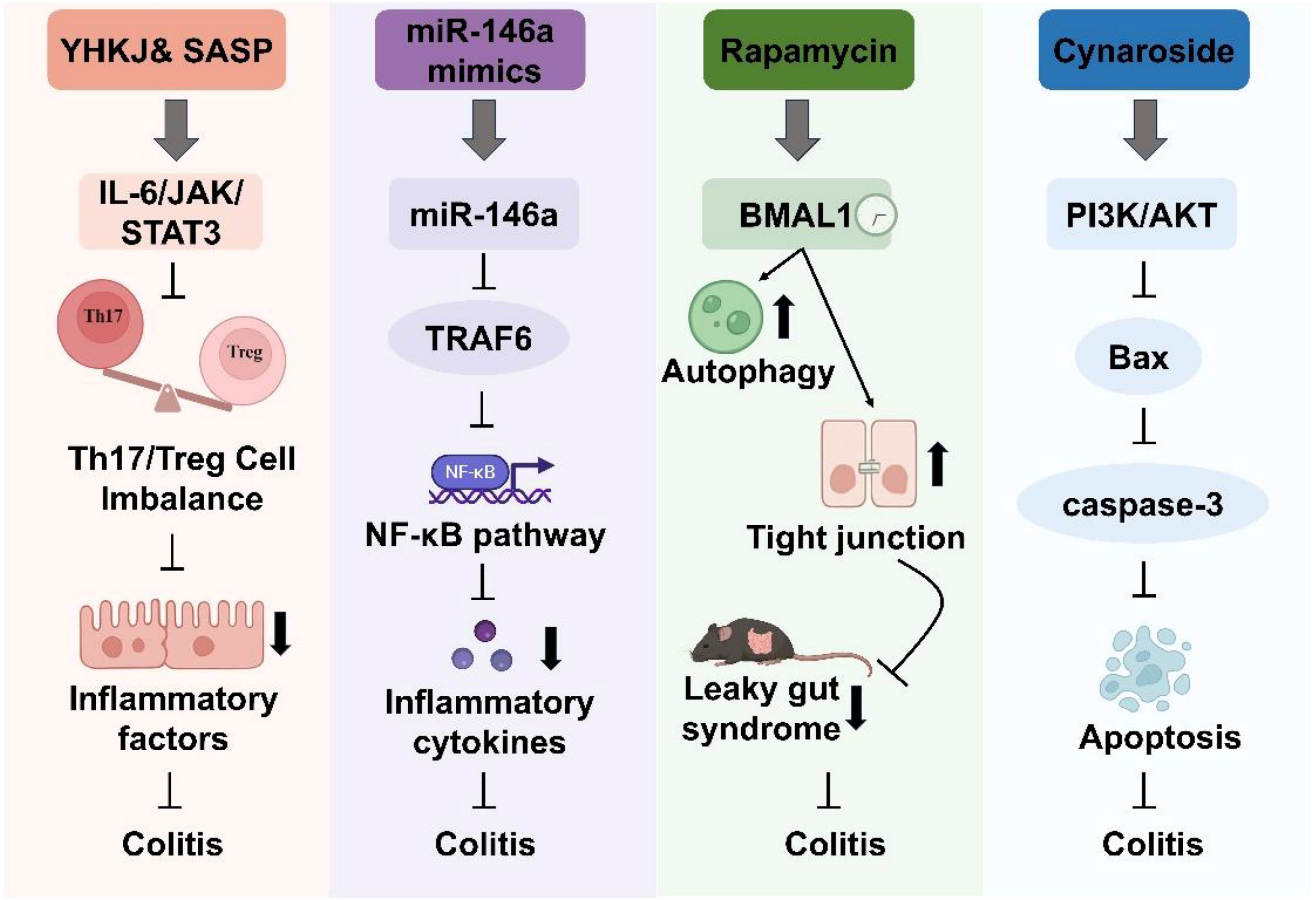
Management strategies for inflammatory bowel disease in this Research Topic. Key targets include miR-146a, BMAL1, essential immune cells, and inflammatory factors. Therapeutic agents encompass both natural compounds, including miR-146a mimics, Youhua Kuijie (YHKJ), sulfasalazine (SASP) and Cynaroside, and synthetic agents like rapamycin.

Building on these mechanistic insights, a diverse array of therapeutic interventions is currently under investigation. One promising therapeutic approach involves the use of miR-146a mimics, synthetically engineered RNA molecules designed to replicate the function of endogenous miR-146a, to downregulate NF-κB-mediated pro-inflammatory gene expression. Preclinical studies have demonstrated that administration of miR-146a mimics can effectively attenuate inflammatory responses and alleviate colitis symptoms in animal models (Zhu et al.) (**Figure 1**). Concurrently, natural bioactive compounds present unique advantages. For instance, the flavonoid Cynaroside modulates the PI3K/AKT pathway, thereby reducing IEC apoptosis and enhancing the expression of tight junction proteins, which fortify the intestinal barrier (Huang et al.) (**Figure 1**). Additionally, rapamycin, an established autophagy inducer, promotes the clearance of damaged cellular components and restores cellular homeostasis, subsequently reducing mucosal inflammation (Zhang et al.) (**Figure 1**). Complementing these modern pharmacological strategies, traditional formulations are also being revisited. The traditional Chinese medicine formulation Youhua Kuijie (YHKJ) has shown significant inhibition of the IL-6/JAK2/STAT3 pathway and restoration of the Th17/Treg balance when used in conjunction with sulfasalazine (SASP) (Tang et al.) (**Figure 1**).

Although therapeutic strategies for intestinal inflammation are diverse, they are all fundamentally aimed at modulating inflammatory signaling and restoring immune balance to support intestinal barrier repair. Therapeutic approaches, including miR-146a mimics, Cynaroside, rapamycin, and the traditional formulation YHKJ, converge on common objectives such as suppressing pro-inflammatory pathways, reducing intestinal epithelial cell apoptosis, and re-establishing the balance between pro-inflammatory and regulatory immune responses. Nevertheless, these strategies exhibit substantial mechanistic heterogeneity. For example, miR-146a mimics provide a targeted, gene-specific intervention but face significant challenges related to *in vivo* delivery and stability. Cynaroside primarily acts through inhibition of the PI3K/AKT pathway to enhance barrier integrity, although its efficacy may vary between individuals. Rapamycin, in contrast, exerts broader effects by promoting autophagy, potentially benefiting a wider patient population. YHKJ, a multi-component traditional Chinese medicine formula, appears to function through complex and synergistic mechanisms that have yet to be fully characterized. This mechanistic diversity suggests the potential value of integrative strategies that combine multiple therapeutic modalities to address individual limitations and advance personalized treatment approaches.

In addition, several bioactive compounds and therapeutic strategies have shown promise in modulating inflammatory responses and restoring immune balance in digestive tract diseases. Ginseng and its active constituents, ginsenosides, exert anti-colitic effects by inhibiting the TLR4/NF-κB signaling pathway and promoting gut microbiota homeostasis (Zhao et al.). Similarly, mannose alleviates IBD symptoms in murine models by strengthening the intestinal immune barrier through microbiota-mediated mechanisms (Yang et al.). Andrographolide demonstrates a protective effect on ulcerative colitis by activating the Nrf2/HO-1-mediated antioxidant response (Shu et al). Atractylenolide I suppresses the release of pro-inflammatory mediators, while Platycodin D activates intestinal AMPK and downregulates genes involved in lipid absorption, thereby indirectly attenuating systemic inflammation (Chen et al.,Tang et al.). Emerging computational approaches have also contributed to the identification of novel biomarkers. A machine learning analysis of large-scale IBD-related datasets revealed *MIR4435-2HG* as a potential diagnostic marker, detectable through a minimally invasive blood test (Stemmer et al.). Beyond pharmacological interventions, dietary strategies such as time-restricted feeding have gained attention for their immunometabolic effects. This approach has been shown to reduce systemic levels of pro-inflammatory cytokines, including IL-6 and TNF-α, activate thermogenic pathways in brown adipose tissue, and reshape the gut-liver axis by enhancing Treg-mediated immunoregulation (Gong et al.). In addition to the aforementioned studies, other contributions within this Research Topic have expanded our understanding of digestive diseases through the identification of novel molecular targets and comprehensive reviews (Wang et al.,Wang et al., Huang et al., Wang et al., Alhendi and Naser).

In summary, recent advances in the identification of therapeutic targets of digestive diseases have driven the development of innovative treatment strategies aimed at modulating inflammation and immune dysregulation. These emerging approaches, encompassing both modern pharmacological agents and traditional medicinal formulations, not only deepen our mechanistic understanding of digestive diseases but also offer promising avenues for precise and effective therapeutic intervention.

